# Immunotherapy protocol with extensively heated milk/egg: preliminary results

**DOI:** 10.1186/2045-7022-3-S3-P34

**Published:** 2013-07-25

**Authors:** F Lazzarotto, R Bonaguro, A Toniolo, MA Muraro

**Affiliations:** 1 Department of Pediatrics-University of Padua, Italy; 2 Food Allergy Centre-Veneto Region, Padua, Italy

## Background

A suggested approach to oral immunotherapy for milk and egg allergy has proposed protocols based on extensively heated milk and egg.

## Methods

Among patients (pts) who attended the Food Allergy Centre in Padua in the last 16 months, 44 cow's milk (CM) and 60 egg (E) allergic pts were recruited. Median age was 9 yrs for CM and 8 yrs for E pts. All pts had reported severe allergic reactions (anaphylaxis), were on a restricted diet for CM or E, and had been prescribed self-injectable epinephrine. All pts underwent an oral food challenge (OFC) for milk/egg before entering the study, to define the threshold of tolerated protein dose. Median CM protein dose was 0,031 g (range 0,00042-1,65); median E protein dose was 0,49 g (range 0,015-3). Up-dosing was scheduled at a 4-6 week intervals and performed in the hospital, under medical supervision. After discharge, pts were requested to daily consume the new tolerated dose for at least one month before the next up-dosing step. Any adverse event or symptom appearing at home could be reported through a 24-hour on-call service. Specific IgE were screened at recruitment: mean value 7.7 kU/L for casein, 3.8kU/L for egg white and 2,3kU/L for ovomucoid. Another blood sample has been scheduled after 12 months to evaluate a change in specific IgE levels. Specific IgG4 to milk/egg proteins will also be screened in both samples.

## Results

Ten drop out were reported: 4 pts for non compliance to the protocol and 6 pts for mild but persistent symptoms induced by the food. Adverse events: 26 pts (25%) developed mild symptoms, requiring no medical treatment or only H1 antagonists; one patient reported wheezing; 3 pts (2,9%) had anaphylaxis (1 at home, 2 in the hospital) requiring epinephrine administration. Over a 12 month period, all pts could increase the tolerated protein dose, and 63,6% of pts could also tolerate less extensively cooked foods.

## Conclusion

Preliminary results from our study show that the daily administration of extensively cooked foods containing milk/egg, can induce an increase of the reactivity threshold, as pts can eat much higher milk/ egg proteins amounts. The protocol appears to be 1) safe, since up-dosing is performed under medical supervision and a constantly monitored consumption at home is guaranteed; 2) well accepted by children, who can expand their diet, and 3) desirable to increase the threshold of reaction, thus reducing the risk of severe reactions from accidental exposure.

## Disclosure of interest

None declared.

